# Pimobendan oral solution is bioequivalent to pimobendan chewable tablets in beagle dogs

**DOI:** 10.1111/jvim.17248

**Published:** 2025-01-21

**Authors:** Olaf Kuhlmann, Michael Markert

**Affiliations:** ^1^ Boehringer Ingelheim Vetmedica GmbH, Clinical Ingelheim Germany; ^2^ Boehringer Ingelheim Pharma GmbH & Co. Ingelheim Germany

**Keywords:** bioequivalence, dog, pharmacodynamic, pharmacokinetics, pharmacology, RSABE

## Abstract

**Background:**

Myxomatous mitral valve disease (MMVD) is frequently diagnosed in small breed dogs. Pimobendan oral solution has been developed to improve dosing accuracy in small and toy breed dogs.

**Hypothesis/Objectives:**

Demonstrate bioequivalence of pimobendan oral solution with pimobendan chewable tablets using a pharmacokinetic and a pharmacodynamic study in healthy purpose bred dogs.

**Animals:**

In the pharmacokinetic study, 24 beagle dogs were dosed in a 4‐period crossover design. In the pharmacodynamic study, 4 mongrel and 2 beagle dogs implanted with telemetry probes were included in a 2‐way crossover design.

**Methods:**

Both studies were designed as prospective, randomized crossover trials. Dogs were given single doses of 5 mg/dog of either formulation followed by serial blood sampling for determination of pimobendan and O‐desmethyl‐pimobendan (ODMP; main metabolite). Because of high variability in the pharmacokinetics, the reference scaled average bioequivalence (RSABE) method was applied. For the pharmacodynamic study, animals were dosed with 0.25 mg/kg of either formulation. Baseline corrected left ventricular maximal pressure (LVdP/dt_max_) and heart rate were recorded continuously and compared with a predefined bioequivalence threshold.

**Results:**

Pimobendan was verified as a high variability drug. Based on the RSABE method, both formulations were bioequivalent. Pharmacodynamic results supported bioequivalence.

**Conclusions and Clinical Importance:**

The novel oral solution of pimobendan was found to be bioequivalent, both applying the Food and Drug Administration (FDA) supported RSABE method and based on pharmacodynamic data. Thus, the novel liquid formulation can be used to facilitate accurate dosing of small and toy breed dogs.

**Abbreviations**
AUC: area under the curve
BE: bioequivalence
EMA: European Medicines Agency
FDA: Food and Drug Administration
GLP: Good Laboratory Practices
MMVD: myxomatous mitral valve disease
ODMP: O‐desmethyl‐pimobendan (main metabolite)
PDE3: phosphodiesterase 3
RSABE: reference scaled average bioequivalence

## 1 INTRODUCTION

Pimobendan is clinically efficacious and safe in the treatment of congestive heart failure in dogs secondary to myxomatous mitral valve disease (MMVD) and in delaying the onset of heart failure in dogs with preclinical heart disease.^1^, ^2^, ^3^, ^4^, ^5^, ^6^ Vetmedin (pimobendan) Chewable Tablets are Food and Drug Administration (FDA) approved for the management of clinical signs of mild, moderate, or severe congestive heart failure in dogs caused by clinical myxomatous mitral valve disease (MMVD) or dilated cardiomyopathy (DCM). Its pharmacodynamic activity is driven by the unique dual mechanism pharmacology of pimobendan. It exerts an inotropic effect by a dual mechanism of action consisting of calcium sensitization and inhibition of phosphodiesterase 3 (PDE3). This effect is combined with vasodilatory activity, also mediated by PDE3 inhibition.^7^ Pimobendan is categorized as an ino‐dilator. The drug undergoes metabolic degradation leading to the formation of an active metabolite, O‐desmethyl pimobendan (ODMP),^8^ which has been shown to reach a similar exposure as does pimobendan in patient dogs.^9^ O‐desmethyl pimobendan also acts as a PDE3 inhibitor and therefore can be expected to substantially contribute to the pharmacology.

In dogs with MMVD, treatment must be continued lifelong after diagnosis, requiring an easy dosing regimen. Although chewable tablets are acceptable for many dogs, the exact dose based on individual dog body weight can be cumbersome because tablets must be divided, and often >1 tablet needs to be administered per dose. Accurate dosing is especially difficult in small and toy breed dogs. Also, toy breed and small dogs may not be willing to chew tablets because of dental disease or taste preferences. A liquid dosing formulation therefore could substantially improve compliance, because it enables accurate dose adjustment based on the dog's body weight.

Our objective was to determine the pharmacokinetics of pimobendan administered using the product Vetmedin (pimobendan) Chewable Tablets in comparison to a newly developed liquid dosing formulation, Vetmedin (pimobendan oral solution) 1.5 mg/mL. However, pimobendan is a drug with highly variable pharmacokinetics.^9^ Highly variable drugs are defined as those for which within‐subject variability (% coefficient of variation [CV]) in bioequivalence (BE) is ≥30%. For such drugs, the FDA developed a reference scaled average bioequivalence (RSABE) approach, whereby the BE acceptance limits are scaled to the variability of the reference product.^10^ For an acceptable RSABE study, a highly variable drug product must meet the scaled BE limit and a point estimate constraint for the geometric mean ratio of the test and reference values for *C*_max_ and area under the curve (AUC).^11^ Because of the high variability of pimobendan, the RSABE method was used in addition to standard BE measures.^11^, ^12^ To support BE based on pharmacokinetic data, the pharmacodynamic effect of both formulations was compared to establish pharmacodynamic BE.

## 2 MATERIALS AND METHODS

### 2.1 Animals

For the pharmacokinetic BE study, 12 male and 12 female healthy adult purpose‐bred beagle dogs were selected that had been bred at WOBE Kereskedelmi Kft., in Budapest, Hungary. Animals had a body weight range of 9.0 to 16.5 kg and an age range of 1 to 10 years at study initiation. For the pharmacodynamic BE study, 2 male and 2 female mongrel dogs and 2 male beagle dogs were used. The dogs had been purpose‐bred by Boehringer Ingelheim Pharma GmbH & Co. KG, Biberach, Germany. The animals had been surgically implanted with telemetry transducers to allow continuous recording of heart rate and left ventricular blood pressure while conscious. For both studies, the animals were drug free for at least 1 month before study start and no medication other than study medication was allowed during the entire study period. Both studies were conducted in compliance with Good Laboratory Practices (GLP; OECD, Paris, 1998; EC Directive 2004/10/EC, 11 February 2004).

### 2.2 Pharmacokinetic bioequivalence study

A replicate crossover study design using 4 periods with full replication (ie, each subject receives the test and reference products twice) was selected for the pharmacokinetic BE study. This design was selected to account for the high inter‐ and intra‐animal pharmacokinetic variability of pimobendan as recently verified in patient dogs, and consistent with the requirements for the RSABE method for BE determination.^9^, ^11^, ^13^ An important benefit of a full replicate design is that it corresponds to the 3 R principles of animal welfare by decreasing the number of experimental animals required. Based on unpublished pilot study data, a sample size of 24 healthy animals (12 male and 12 female animals) was estimated to be sufficient to reach the objective of the study.

Animals were sorted by body weight and randomly allocated to 1 of 2 treatment groups using a computerized randomization procedure (SAS software release 9.4, Cary, North Carolina, USA). Until the final statistical evaluation was performed, the responsible veterinarian, the sponsor representative, the bioanalyst, and all responsible personnel were masked, except the study director overseeing all study tasks to ensure the protocol was followed and the personnel administering the formulation. The responsible veterinarian was the only person in charge of potentially removing animals from the study.

Both the test product Vetmedin (pimobendan oral solution) 1.5 mg/mL (A; approved; market entry expected soon) and the reference product Vetmedin (pimobendan) Chewable Tablets (B; pioneer product by Boehringer Ingelheim, imported from the United States) were dosed at 5 mg/dog, regardless of the body weight of the animals, following the treatment design as described in Table 1. The animals were fasted for at least 12 hours before each treatment to prevent a food effect with drug absorption, consistent with the prescribing information for pimobendan chewable tablets. Food was offered again after blood collection 4 hours after treatment.

**TABLE 1:** Experimental design.

Group	No. of animals	Target dose (mg/animal)	Route	Treatment^a^			
				Day 0	7	14	21
I	12	5	Oral	A	B	A	B
II	12	5	Oral	B	A	B	A

^a^ A = pimobendan 1.5 mg/mL oral solution (test product). B = pimobendan 5 mg chewable tablets (reference product).

Before inclusion and again on the day before study start, all animals underwent a complete physical examination to ensure good health status, and this examination was repeated on the last study day. All animals were handled regularly and observed daily over the entire time period of the study. These observations included general physical appearance and behavior, posture, food and water consumption, appearance of urine and feces, skin and coat, condition of body orifices and any signs of ill health. On dosing days, additional observations were added before each blood collection. In addition, body weight was recorded once weekly on the day before each dose.

For each treatment period, 16 blood samples were drawn either via the cephalic or saphenous vein at 0 hour (ie, before treatment; baseline), 15, 30 and 45 minutes after treatment with an acceptable variance of ±5 minutes, as well as at 1, 2, 3, 4, 5, 6, 7, 8, 9, 10, 11 and 12 hours with an acceptable variance of ±10 minutes after treatment, collecting a volume of 3 mL/sample in K3‐EDTA tubes. Samples were immediately cooled in ice water. Within 60 minutes after collection, samples were centrifuged and plasma was harvested and frozen at −20°C for later analysis.

### 2.3 Pharmacodynamic study

To support the BE determination based on a pharmacodynamic endpoint, a pharmacodynamic study was conducted in 2 male and 2 female healthy mongrel dogs and 2 male beagle dogs. The aim was to evaluate the effect of pimobendan on maximal left ventricular contractility (LVdP/dt_max_) and heart rate. The parameter LVdP/dt_max_ is sensitive for the PDE3 inhibition‐mediated effect of pimobendan and its active metabolite, whereas heart rate is not expected to change after pimobendan administration in healthy dogs at the dose given. After a crossover 2‐period design all animals were randomly assigned to receive a single dose of either formulation, with a 48 hour washout between doses. Based on the known terminal half‐life of 0.97 hours for pimobendan and 1.33 hours for ODMP,^9^ this washout period is sufficient. Animals previously had been implanted with a telemetry device with 2 high fidelity pressure transducers and ECG cables capable of continuously recording left ventricular pressure and heart rate (“T27” total implant, Integrated Telemetry Services, Pinckney, Michigan or “L21” total implant, DSI, Data Sciences, Indianapolis, IN). On each experimental day, the overnight‐fasted animals were allowed an initial equilibrium period of 120 minutes to acclimate to their measurement pens which were equipped with the telemetry radio receivers, after which they were given the oral solution or the chewable tablets at a target dosage of 0.25 mg/kg body weight. Dogs were fed 1 hour after administration and then were left undisturbed for the duration of the study. Recording started 1 hour before dosing for baseline recording and was continued for 7 hours after dosing. Forty‐eight hours after the first administration, the procedure was repeated with the respective alternate formulation. For dose administration, 2 strengths of pimobendan chewable tablets, 2.5 and 5 mg, were utilized and the lower strength tablet could be administered as half or full tablet to achieve a dose best matching the target dosage of 0.25 mg/kg. The dose of the oral solution was adjusted to match the tablet dose for the respective dog. Based on the individual body weights of the animals, the actual dose administered ranged from 0.25 to 0.32 mg/kg for both formulations. The telemetry signals were recorded and processed using a fully validated computer system (Notocord Hem software version 3.5, Instem, Stone, Staffordshire, UK).

### 2.4 Plasma and metabolite analysis

All plasma samples were analyzed according to GLP for concentrations of pimobendan and its active metabolite ODMP using a fully validated liquid chromatography‐mass‐spectrometry (LC‐MS/MS) assay that was validated according to international guidelines and approved by the Center for Veterinary Medicine (CVM). In brief, the analytes were extracted from dog plasma after addition of the internal standard [^2^H_3_]‐pimobendan (deuterated pimobendan), using supported liquid extraction (SLE) followed by chromatographic separation on a Phenomenex Luna Omega Polar C18 column (50 × 2.1 mm, 1.6 μm) and mass spectrometric detection using positive multiple‐reaction monitoring on a Sciex API5000 instrument. All plasma samples were stored at −20°C ± 5°C and were analyzed within 49 days after sample collection, which is within the documented stability for the analytes of 392 days at −20°C ± 5°C. The method validation and analysis of the study samples were performed in compliance with European Medicines Agency (EMA) and FDA requirements. The assay performance data are summarized in Table 2.

**TABLE 2:** Bioanalytical assay performance data.

Item	Unit	Pimobendan	O‐desmethyl‐pimobendan metabolite
Calibration range	ng/mL	0.100‐50.0	0.100‐50.0
Defined LLOQ	ng/mL	0.100	0.100
Required plasma volume	μL	50.0	50.0
QC accuracy	%	98.5‐103.2	96.0‐100.4
QC precision	%	4.8‐6.8	4.0‐7.8
ISR samples measured	N	128	128
ISR pass rate	%	93.0	82.0

Abbreviations: ISR, incurred sample reanalysis; LLOQ, lower limit of quantification; QC, quality control samples.

### 2.5 Statistical methods, bioequivalence

Because pimobendan was expected to be a highly variable drug with large intraindividual variability in pharmacokinetics based on data generated in patient dogs,^9^ the RSABE approach was selected. For this procedure, a 2‐step analysis is performed. In the first step, using logarithmically transformed data, the within‐subject standard deviation (SWR) of the reference product pimobendan chewable tablets is determined for *C*_max_, AUC_0➔t_ and AUC_last_. If SWR is <0.294 as determined for each parameter independently, the RSABE is not applicable, and standard average based BE criteria apply. If SWR is ≥0.294, the BE criteria are widened for the affected parameter, depending on the individual SWR as determined for the reference product. The threshold of 0.294 was selected based on the FDA draft guidance on progesterone, describing the RSABE approach in detail.^11^, ^13^

The following equation was applied to each parameter based on the widened BE criteria if SWR was ≥0.294 (ie, if high variability was verified):

Bioequivalence limit:

$$\frac{{\left(\mu_{T}-\mu_{R}\right)}^{2}}{\sigma_{\mathrm{WR}}^{2}}\leq \frac{{\left(\ln\left(1.25\right)\right)}^{2}}{\sigma_{\mathrm{WO}}^{2}},$$

(1) $$\frac{{\left(\ln\left(1.25\right)\right)}^{2}}{\sigma_{\mathrm{WO}}^{2}}=\theta_{S},$$

*μ*_*T*_ = average response of the log‐transformed measure for the test (T) formulation; *μ*_*R*_ = average response of the log‐transformed measure for the reference (R) formulation; *σ* ^2^_*WR*_ = within‐subject variance of the reference formulation; *σ* ^2^_*W*0_ = predetermined constant set by the regulatory agency. *σ*_*W*0_ is set at 0.25^11^, ^13^; *θ*_*S*_ = adjusted bioequivalence limit.

If this equation was found to be true, the first BE criterion was fulfilled.

Equation (1) can be simplified by focusing on the upper confidence bound in relation to the adjusted BE limit based on Howe's Approximation I to approximate confidence limits as in Equation (2)^14^:

(2) $${\left(\mu_{T}-\mu_{R}\right)}^{2}-\theta_{S}\;{\sigma^{2}}_{\mathrm{WR}}\;\leq 0.$$

Taking this equation into account, the test and reference product are concluded to be bioequivalent if:

1. the 95% upper confidence bound for (*μ*_*T*_ − *μ*_*R*_)^2^ − *θ*_*S*_ *σ* ^2^_*WR*_ is ≤0; and
2. the test/reference geometric mean ratio (GMR) in the study falls within [0.8, 1.25].

Because the RSABE approach is not accepted by all regulatory agencies, in addition, a standard BE evaluation was performed. According to this approach, both treatments are accepted as bioequivalent if the antilog of the 90% confidence interval of the difference between the 2 treatments for *C*_max_ and AUC_0➔t_ is entirely contained within the limits of 80% to 125%. In addition, the EMA may allow for *C*_max_ widened limits of 70% to 143%, if the widening can be justified (eg, pharmacodynamic data of biological equivalence).^15^

All calculations were performed for pimobendan and ODMP using the validated SAS software release 9.4 (Cary, North Carolina, USA: SAS Institute Inc, 2013). For the RSABE, the method for statistical analysis followed the example SAS code as outlined in the FDA's Draft Guidance on Progesterone.^13^

For the statistical evaluation of the pharmacodynamic study, the recording was divided into 2 time intervals, 60 minutes to 20 minutes before dosing as baseline interval, and 30 minutes post dosing until 7 hours as treatment phase. Both parameters, LVdP/dt_max_ and heart rate, were baseline adjusted using the median of the predose values for each animal and each period. Within the observation phase, the measured values for an animal were aggregated to the standardized area under the curve (AUC divided by interval length). The percentage of the standardized AUC compared to individual baseline was calculated for each animal. This percentage of baseline was used as a target variable for the statistical evaluation. Baseline‐adjusted mean differences between both treatments with a 2‐sided 90% confidence interval were calculated. Pharmacodynamic BE was accepted if the 90% confidence interval of the percentage difference between the 2 treatments for LVdP/dt_max_ and heart rate was entirely contained within the limits of 80% to 120%.

## 3 RESULTS

### 3.1 Pharmacokinetic bioequivalence study

**FIGURE 1:**
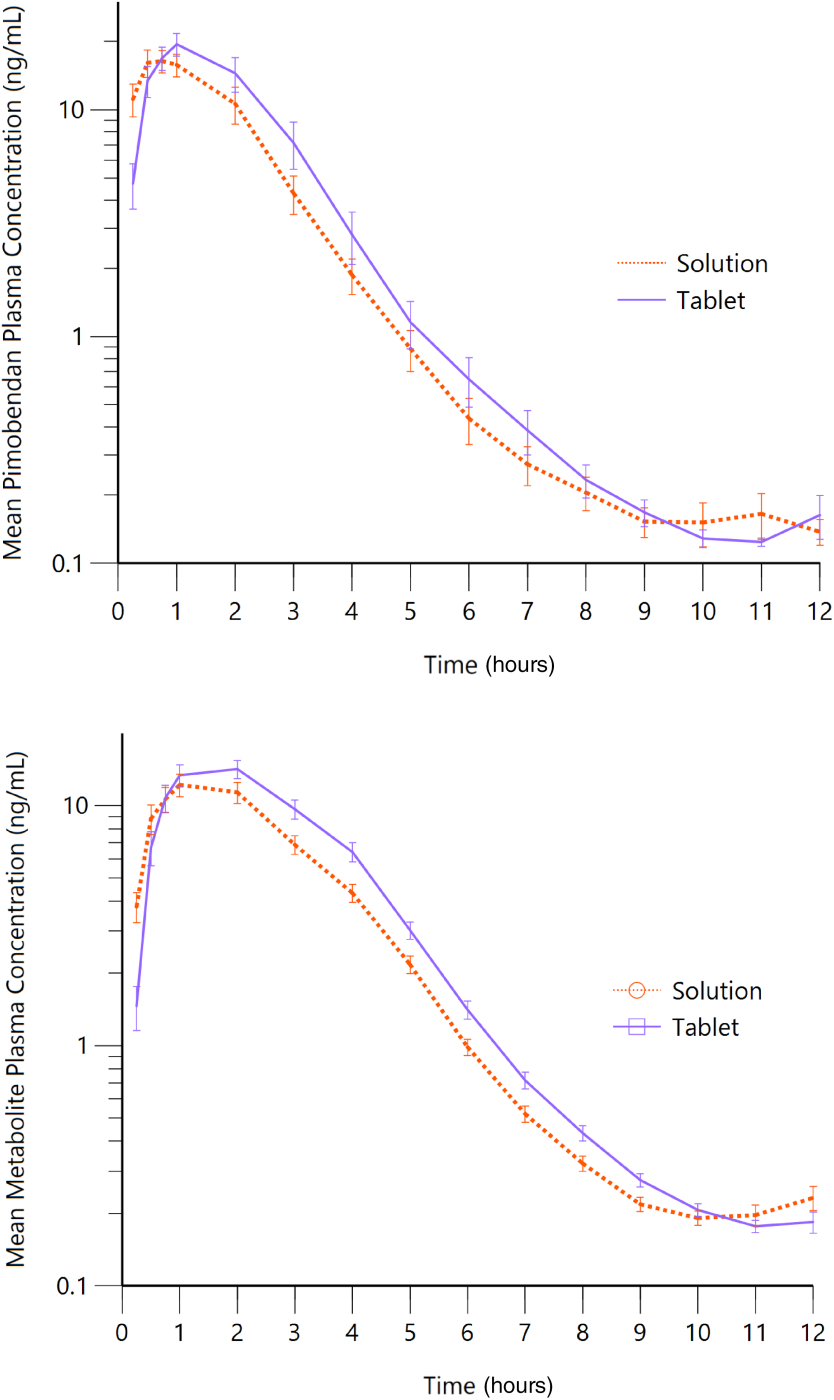
Pharmacokinetics of pimobendan and the O‐desmethyl pimobendan (ODMP) following oral administration of a single 5 mg dose to 12 male and female beagle dogs following a 4‐period crossover design (each formulation administered twice). Displayed are mean ± SE data for pimobendan oral solution (red dotted line) and pimobendan chewable tablets (blue solid line).

Pimobendan, administered as a chewable tablet or as an oral solution at a fixed dose of 5 mg/dog, single administration, during a 4‐period crossover study, was well tolerated. Clinical signs attributable to pimobendan were not noted during the entire course of the study and no drug related adverse events were recorded. The plasma concentration‐time course is presented in Figure 1 for pimobendan and its metabolite ODMP. Pharmacokinetic parameters calculated by noncompartment analysis are presented in Table 3. Peak plasma concentrations and exposures, as expressed by AUC for both pimobendan and ODMP, were highly variable. In contrast, the terminal half‐life and mean residence time were relatively homogenous. The SWR for pimobendan chewable tablets was 0.601 for *C*_max_, 0.417 for AUC_last_, and 0.415 for AUC_last_ (Table 4). The SWR for ODMP after administration of pimobendan chewable tablets was 0.451 for *C*_max_, 0.385 for AUC_last_, and 0.382 for AUC_last_. All 6 parameters exceeded the FDA‐defined threshold of 0.294 for highly variable drugs. Thus, the RSABE approach for BE calculation is applicable to pimobendan and its metabolite ODMP. The point estimates comparing both formulations were 0.927 for *C*_max_, 0.853 for AUC_0➔t_, and 0.855 for AUC_last_ for pimobendan with 95% upper confidence bounds of −0.153 for *C*_max_, −0.029 for AUC_0➔t_, and −0.030 for AUC_last_. For ODMP, the point estimates were 0.901 for *C*_max_, 0.825 for AUC_0➔t_, and 0.827 for AUC_last_ with 95% upper confidence bounds of −0.054 for *C*_max_, 0.017 for AUC_0➔t_, and AUC_last_. The data indicate BE of the test product pimobendan 1.5 mg/mL oral solution with the reference product pimobendan 5 mg chewable tablets based on the plasma concentrations of the parent compound pimobendan because the point estimates were within [0.80, 1 .25] and the 95% upper confidence bounds (Equation 2) were <0. Regarding the metabolite ODMP, BE criteria were met for *C*_max_ but not for AUC_0➔t_ and AUC_last_.

**TABLE 3:** Pharmacokinetic parameter for pimobendan and its O‐desmethyl pimobendan metabolite (ODMP).

Parameter		Oral solution 1.5 mg/mL		Chewable tablets 5 mg	
		Pimobendan	ODMP	Pimobendan	ODMP
*T*_max_ (hour)	Mean ± SD	1.005 ± 0.679	1.333 ± 0.711	1.365 ± 0.908	1.714 ± 0.899
*C*_max_ (ng/mL)	Mean ± SD	23.9798 ± 18.3250	16.0423 ± 9.8588	25.6619 ± 19.6540	17.2623 ± 9.2246
AUC_0➔t_ (ng•h/mL)	Mean ± SD	38.7731 ± 26.2055	40.3817 ± 20.3112	46.6520 ± 38.9429	49.1545 ± 24.2688
AUC_0➔∞_ (ng•h/mL)	Mean ± SD	38.9405 ± 26.1857	40.6725 ± 20.3486	46.7979 ± 38.9302	49.4346 ± 24.2638
*T*_1/2_ (hour)	Mean ± SD	0.804 ± 0.270	1.596 ± 0.516	0.736 ± 0.239	1.650 ± 0.449
MRT (hour)	Mean ± SD	1.741 ± 0.675	2.627 ± 0.584	1.950 ± 0.747	2.840 ± 0.578

Abbreviations: AUC_0➔t_, area under the plasma concentration time curve until last sampling time point; AUC_last_, area under the plasma concentration time curve extrapolated to infinity; *C*_max_, maximal plasma concentration; MRT, median residence time; *T*_max_, time of maximal plasma concentration; *T*_1/2_, terminal half‐life.

**TABLE 4:** Results of crossover ANOVA and reference‐scaled average bioequivalence approach for pimobendan and its O‐desmethyl pimobendan metabolite (ODMP).

Parameter	Effect	Pimobendan/ODMP
*C*_max_	Test/reference	Point estimate: 0.927/0.901 SWR: 0.601/0.451 Critical bound: −0.153/−0.054
AUC_0➔t_	Test/reference	Point estimate: 0.853/0.825 SWR: 0.417/0.385 Critical bound: −0.029/0.017
AUC_0➔∞_	Test/reference	Point estimate: 0.855/0.827 SWR: 0.415/0.382 Critical bound: −0.030/0.017

In addition to the RSABE approach, data were analyzed following the standard BE criteria (Tables 5 and 6). The point estimates were 0.901 for *C*_max_, 0.825 for AUC_0➔t_, and 0.827 for AUC_last_. The antilogs of the 90% confidence intervals for the difference between the 2 formulations were 0.762 to 1.064 for *C*_max_, 0.718 to 0.949 for AUC_0➔_t, and 0.720 to 0.949 for AUC_last_. The confidence interval for *C*_max_ was not entirely contained within the BE range of 0.8 to 1.25, but it was within the wider range of 0.7 to 1.43. For AUC_0➔t_ and correspondingly for AUC_last_, the confidence intervals were not within the BE range of 0.8 to 1.25. Thus, applying standard BE criteria, including the widened acceptance criteria for *C*_max_, and based on the pharmacokinetics of pimobendan, both formulations were not bioequivalent, because the confidence intervals for AUC exceeded the predefined limits.

**TABLE 5:** Results of crossover analysis of variance (ANOVA) and bioequivalence for pimobendan.

Parameter	Effect	Point estimate	95% confidence interval	
*C*_max_	Period 1 Period 2 Period 3 Period 4	14.9922 17.3504 15.8028 27.1095	11.7405 13.5873 12.3753 21.2297	19.1444 22.1558 20.1795 34.6177
	Test Reference	17.5950 18.9726	14.8017 15.9605	20.9155 22.5530
	Sequence 1‐2‐1‐2 Sequence 2‐1‐2‐1	22.9251 14.5614	15.8750 10.0834	33.1061 21.0282
	**Test/reference**	**Point estimate:** **90% confidence interval:**	**0.927** **0.756‐1.138**	
AUC_0➔t_	Period 1 Period 2 Period 3 Period 4	28.5105 32.0790 30.9569 47.2606	24.0240 27.0310 26.0855 39.8236	33.8347 38.0697 36.7380 56.0864
	Test Reference	31.4161 36.8204	27.8339 32.6219	35.4595 41.5592
	Sequence 1‐2‐1‐2 Sequence 2‐1‐2‐1	42.4027 27.2802	31.1211 20.0221	57.7739 37.1694
	**Test/reference**	**Point estimate:** **90% confidence interval:**	**0.853** **0.739‐0.984**	
AUC_0➔∞_	Period 1 Period 2 Period 3 Period 4	28.7256 32.3017 31.1593 47.4371	24.2461 27.2646 26.3003 40.0397	34.0327 38.2695 36.9160 56.2012
	Test Reference	31.6476 37.0049	28.0723 32.8244	35.6782 41.7178
	Sequence 1‐2‐1‐2 Sequence 2‐1‐2‐1	42.6038 27.4885	31.3272 20.2127	57.9395 37.3834
	**Test/reference**	**Point estimate:** **90% confidence interval:**	**0.855** **0.742‐0.985**	

**TABLE 6:** Results of crossover analysis of variance (ANOVA) and bioequivalence for O‐desmethyl pimobendan metabolite (ODMP).

Parameter	Effect	Point estimate	95% confidence interval	
*C*_max_	Period 1 Period 2 Period 3 Period 4	11.3845 13.5624 12.0133 19.2440	9.3257 11.1096 9.8407 15.7638	13.8980 16.5566 14.6656 23.4927
	Test Reference	13.0440 14.4842	11.3279 12.5786	15.0201 16.6784
	Sequence 1‐2‐1‐2 Sequence 2‐1‐2‐1	16.3586 11.5494	12.4579 8.7955	21.4806 15.1657
	**Test/reference**	**Point estimate:** **90% confidence interval:**	**0.901** **0.762‐1.064**	
AUC_0➔t_	Period 1 Period 2 Period 3 Period 4	32.4912 38.0220 36.2946 52.4614	27.4921 32.1719 30.7103 44.3897	38.3994 44.9359 42.8943 62.0009
	Test Reference	35.5806 43.1050	31.6160 38.3020	40.0424 48.5103
	Sequence 1‐2‐1‐2 Sequence 2‐1‐2‐1	45.9168 33.4018	37.6968 27.4223	55.9291 40.6952
	**Test/reference**	**Point estimate:** **90% confidence interval:**	**0.825** **0.718‐0.949**	
AUC_0➔∞_	Period 1 Period 2 Period 3 Period 4	32.8034 38.3403 36.6123 52.8022	27.8071 32.5007 31.0358 44.7598	38.6975 54.2293 43.1908 62.2897
	Test Reference	35.9005 43.4336	31.9416 38.6440	40.3502 48.8170
	Sequence 1‐2‐1‐2 Sequence 2‐1‐2‐1	46.2285 33.7301	37.9932 27.7214	56.2487 41.0413
	**Test/reference**	**Point estimate:** **90% confidence interval:**	**0.827** **0.720‐0.949**	

### 3.2 Pharmacodynamic study

**FIGURE 2:**
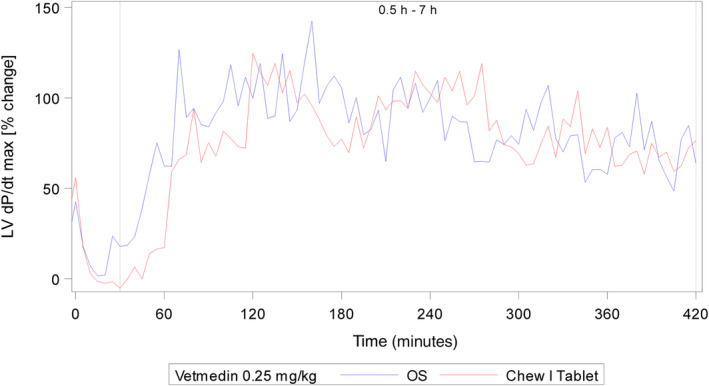
Left ventricular peak pressure (LVdP/dt_max_) following oral administration of pimobendan chewable tablets or pimobendan oral solution at a targeted dose of 0.25 mg/kg. Dotted line denotes start and end of statistical evaluation period.

**FIGURE 3:**
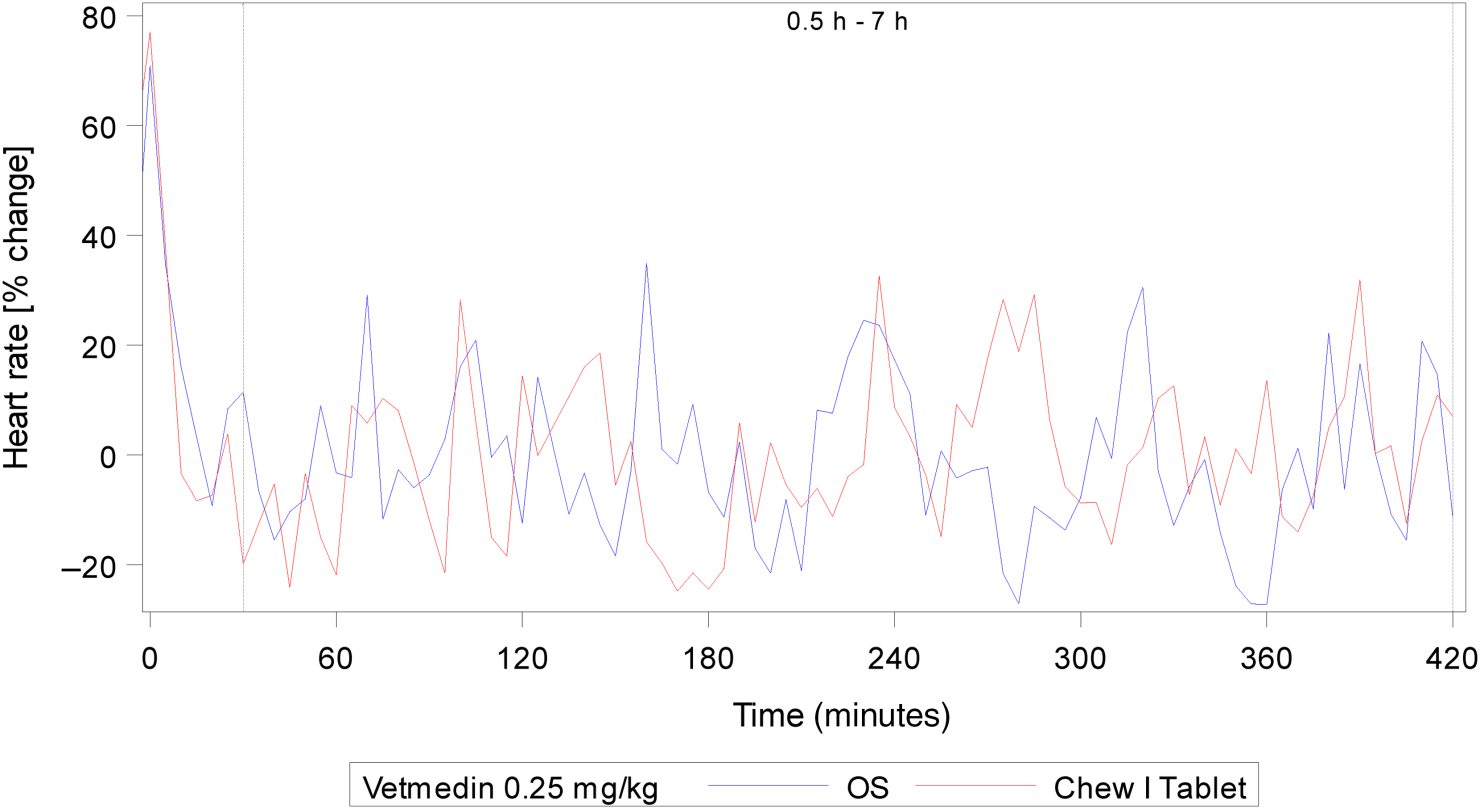
Heart rate following oral administration of pimobendan chewable tablets or pimobendan oral solution at a targeted dose of 0.25 mg/kg. Dotted line denotes start and end of statistical evaluation period.

To support BE, a pharmacodynamic study was conducted in 2 male and 2 female mongrel dogs and 2 male beagle dogs that had telemetry devices surgically implanted for quantification of left ventricular maximal contractility LVdP/dt_max_ and heart rate.^16^ After pimobendan administration, a rapid increase in cardiac contractility was observed, following the expected increase in plasma exposure for both pimobendan and ODMP. The peak for increase in contractility was observed approximately 2 hours after dosing reaching approximately twice the baseline contractile force, with a slightly more rapid increase after administration of the oral solution, consistent with the pharmacokinetic parameter for pimobendan and its metabolite ODMP (Table 3). Using baseline adjusted data, the mean increase for the period 0.5 to 7 hours postdose was on average 84.88% and 79.48% after administration of 0.25 mg/kg pimobendan 1.5 mg/mL oral solution and pimobendan chewable tablets, respectively (Table 7; Figure 2). No significant difference was found between the oral solution and the chewable tablets at 0.25 mg/kg for LVdP/dt_max_. No clinically relevant change (increase or decrease) in heart rate was observed after administration of pimobendan for either formulation (Table 7; Figure 3). For both parameters, the confidence intervals of the means were well within the predefined range of 80% to 120%, indicating that both formulations are equivalent based on the pharmacodynamic effects.

**TABLE 7:** Pharmacodynamic bioequivalence of pimobendan.

	Pimobendan oral solution 1.5 mg/mL		Pimobendan chewable tablets	
Adjusted mean	Heart rate	LVdP/dt_max_	Heart rate	LVdP/dt_max_
Adjusted mean (% of baseline)	97.63	184.88	101.28	179.48
Mean difference			3.66	−5.40
*P*‐value (difference)			.4540	.4210
Lower confidence limit (90%)			−5.75	−18.25
Upper confidence limit (90%)			13.07	7.45

*Note*: Mean difference = difference between adjusted mean of both devices; *P*‐value <.05.

## 4 DISCUSSION

The majority of breeds at risk for congestive heart failure caused by MMVD have an average adult body weight of <9 kg.^17^ Exact dosing at a dosage of 0.25 mg/kg 2 times per day (q12h) with oral tablet formulations of 2.5 and 5 mg may be challenging and requires division of tablets. Therefore, a liquid oral formulation is of high interest for easy dose administration for pet owners and for adjustment of doses to the individual body weights, especially for small breeds. We have investigated the pharmacokinetics and pharmacodynamics of 2 pimobendan formulations, the marketed originator product chewable tablets, and a novel oral solution with a drug concentration of 1.5 mg/mL, to evaluate biological equivalence of both formulations. The plasma concentration analysis indicated high variability of pimobendan (and its metabolite ODMP) exposure. Such variability has been reported previously in patient dogs.^9^ Although the authors demonstrated that the pharmacokinetics of pimobendan in patient dogs and healthy dogs were similar, they failed to identify the potential cause of the variability. Dog characteristics such as age, breed, body condition score, mg/kg dose, stage of heart disease, or liver function were not found to be linked to the differences. Comparing both studies, it can be seen that the AUC ratio between pimobendan and ODMP (1:1.07 in patients, 1:1.05 in our study) as well as the terminal half‐life of both drug products (0.97 and 1.33 hours in patients, 0.74 and 1.65 hours in our study after ingestion of the chewable tablets) are very similar. Furthermore, both factors had only moderate variability in our study. These results suggest that variability is primarily related to absorption and less to metabolic degradation or excretion.

High pharmacokinetic variability generates a high burden for proof of BE of different formulations. Although variability can be partially counterbalanced using larger study populations to generate BE data, doing so violates the principles of 3R that call for a decrease in the number of animals used in animal experiments. Examples exist in which a highly variable reference product failed to demonstrate BE when compared with itself in a BE study using a standard design and sample size, indicating that new methods must be developed.^18^ The FDA has developed a method to adjust the BE analysis of high variability drugs in humans (ie, the RSABE method).^11^, ^13^ The RSABE is only applicable to highly variable drugs, whereas for drugs without high variability, the standard BE approach with a BE limit of 80% to 125% for the geometric mean ± 90% confidence limits are to be applied. Although the RSABE approach is implemented by the FDA in the United States now, it is not yet adopted by EMA. However, EMA recently has updated its approach for veterinary medicinal products proposing an alternate approach, where the limits for the confidence intervals for *C*_max_ were widened to 70% to 143%, whereas no adjustment was made for AUC.^15^ This approach is only applicable if justified (eg, pharmacodynamic data supporting biological equivalence). We have compared both approaches for our data set. Although the RSABE method clearly demonstrated BE of both formulations, and pharmacodynamic data also support the BE, the EMA method failed to demonstrate BE. Although the *C*_max_ values were within the widened range of 70% to 143%, the AUC failed to meet the criterion, which is not widened according to EMA regulations. Our data thus demonstrate that the current EMA approach is still too restrictive for drugs with high variability and does not match the FDA approach. However, our pharmacodynamic data obtained in 6 dogs with telemetry transducers, including 2 breeds and both sexes, clearly demonstrate biological equivalence of both formulations, confirming our RSABE assessment of BE.

In conclusion, our data demonstrate the pharmacokinetic BE of pimobendan oral solution 1.5 mg/mL compared to the originator product pimobendan chewable tablets, applying the FDA criteria for BE for highly variable drugs. The pharmacokinetic data are supported by pharmacodynamic data demonstrating a robust and comparable pharmacodynamic effect on cardiac contractility for both formulations. However, the EMA criterion for plasma concentration‐based BE was not met for 1 of the required criteria, namely the AUC_0➔t_. The failure following pharmacokinetic EMA criteria, despite strong pharmacodynamic data of equivalence indicates that those criteria may be too restrictive, and that the widening of the limits as allowed for *C*_max_ also should be extended to AUC. Because the RSABE approach results in acceptance criteria that are adjusted based on the intraindividual variability of the reference product, this approach is more flexible than the EMA proposal to simply widen the acceptance interval with support of pharmacodynamic or clinical justification.

## CONFLICT OF INTEREST DECLARATION

Dr Olaf Kuhlmann and Michael Markert are both employees of Boehringer Ingelheim, the company which has developed and marketed the originator product as well as the novel oral solution under the brand name Vetmedin (pimobendan oral solution) 1.5 mg/mL. The study was fully funded by Boehringer Ingelheim, Germany.

## OFF‐LABEL ANTIMICROBIAL DECLARATION

Authors declare no off‐label use of antimicrobials.

## INSTITUTIONAL ANIMAL CARE AND USE COMMITTEE (IACUC) OR OTHER APPROVAL DECLARATION

The pharmacokinetic study in 24 beagle dogs, conducted by BioMedVet Research GmbH, Germany, on behalf of Boehringer Ingelheim Vetmedica GmbH, was approved by the Lower Saxonian state institution on consumer protection and food safety, Oldenburg (File number 33.19‐42 502‐05‐19A462, 21st October 2019).

The pharmacodynamic study in 6 mongrel and beagle dogs conducted by Boehringer Ingelheim, Biberach, Germany, followed the German Law on the Protection of Animals and was performed with permission of the regional authorities. All animals were held in an Association for Assessment and Accreditation of Laboratory Animal Care (AAALAC) International accredited facility for the duration of the study.

## HUMAN ETHICS APPROVAL DECLARATION

Authors declare human ethics approval was not needed for this study.
